# The health-related benefits and complications of tube-fed blended diet for children and young people

**DOI:** 10.12968/gasn.2025.0021

**Published:** 2025-07-02

**Authors:** K Ujhelyi Gomez, C Ingleby, J Hill

**Affiliations:** University of Liverpool; Lancashire Teaching Hospitals NHS Foundation Trust; University of Lancashire

**Keywords:** blended diet, children and young people, systematic review, tube feeding, gastrointestinal symptoms

## Abstract

**Background:**

Research on blended diet for tube-fed children has been identified as a top priority for clinicians and families due to its growing popularity.

**Aim:**

This article evaluates and summarises a systematic review that examined the benefits and complications of using blended diet in children and young people and expand upon its findings in the context of current evidence and practice.

**Methods:**

The Joanna Briggs Institute Critical Appraisal Checklist was used to critically appraise the review.

**Findings:**

Blended diet may have the potential to lead to positive outcomes.

**Conclusion:**

Currently the evidence regarding the benefits and complications of tube-fed blended diet for children and young people is limited and further better-quality research and reporting is needed in the area.

## Introduction

The largest group of children and young people living with disabilities is those who have neurodevelopmental impairments and conditions (Harris et al. 2017). Eating, drinking and swallowing difficulties are common in this group (Taylor et al. 2021) with up to 85% of children with spastic quadriplegia requiring gastrostomy support (Köglmeier et al. 2023).

The primary source of nutrition for most children and young people using a feeding tube is a commercially produced, nutritionally complete liquid feed (commercial formula, CF, which has been widely used since the 1970’s (Campbell 2006). In recent years, however, there has been a resurgence, particularly within the paediatric population, for families to choose to blend regular family foods (Blended Diet, BD) as an alternative means to nourish their child (Köglmeier et al. 2023; British Dietetic Association (BDA) 2021). Through using BD, families seek to improve feed tolerance and community forums suggest that symptoms such as loose stools, constipation, gastrointestinal (GI) discomfort, reflux and general malaise may be improved (Köglmeier et al. 2023). For many families the ability to include their tube-fed child in regular family meals has been substantially rewarding, increasing wellbeing and family cohesion (Köglmeier et al. 2023).

However, the practice of using BD has been met with caution and scepticism by some healthcare professionals with concerns such as tube failure or blockage, microbial contamination and infection, and nutritional inadequacy (Groucutt et al. 2019). Traditional nursing training and NICE guidance have previously advised that only commercial feeds, water and medications should be given via an enteral feeding tube (Clinical Resource Efficiency Support Team (CREST) 2004; NICE 2017) and, therefore, some nurses have struggled to engage with the practice. However, recently published work and support in the Enteral Product Safety Group publications (British Specialist Nutrition Association (BSNA) 2022) has shown that suitable, appropriate, and safe guidelines regarding the use of BD can be successfully developed and implemented (Thomas 2016; Shovlin et al. 2023).

The disconnect between families’ preference for BD and the reluctance seen in some healthcare professionals has led to different practices around the UK (Doyle et al, 2021). In some areas, all stakeholders work together with families to support nurseries, schools and hospitals (Thomas 2016). Everyone is fully engaged with the practice and clinicians can support the individual needs of the children in their care (Shovlin et al. 2023). In other areas, the child may have to switch to a commercial formula when away from parents or in an acute hospital ward (Bremner et al. 2022). This means the child may not fully benefit from BD to potentially improve symptomatology. As a result, parents/carers tend to resent official recommendations and choose to navigate the practice of BD independently without professional support leading to further conflict and disengagement (Doyle et al. 2021).

Diskin et al (2022) conducted a modified prioritisation Delphi study surveying caregivers and clinicians. All stakeholders agreed that poor feed tolerance was a significant burden impacting the lives of children and families (Diskin et al. 2022). With the emerging evidence supporting the use of BD, this topic was selected as a key research priority. Previous systematic reviews had investigated prevalence, efficacy and safety aspects of BD (Carter et al. 2018) in adult or mixed (adult and child) populations (Brown et al. 2020; Peers et al. 2023) or compared the use of homemade and commercial blends (Bennett et al. 2020). To address the gap in the literature, McCormack et al (2023) sought to explore the clinical outcomes (benefits and complications) associated with BD use by children and young people.

## Aim of commentary

This commentary aims to critically appraise the methods used within the review by McCormack et al (2023) and expand upon the findings in the context of clinical practice.

## Methods of McCormack et al (2023)

The authors undertook a protocol registered systematic review with a comprehensive literature search using a range of electronic databases (PubMed, Embase, CINAHL, Scopus, Cochrane), including the grey literature from inception to January 2021.The search was updated in August 2022. Reference and citation tracking was also performed, and authors were contacted for further information where needed.

Studies met the inclusion criteria if they were original research from randomised control trials (RCT), interventional and observational studies including cohort studies and were available in English. Inclusion criteria regarding age was unclear. Studies were excluded if they were single case reports, had a primary focus on the management, equipment, preparation, or attitudes to enteral feeding or compared commercial blends only.

Titles, abstracts, and full texts were screened by one researcher and uncertainties were discussed with another, who then reviewed all included papers for eligibility. The lead author extracted the data, and a second author checked for accuracy. The Mixed Methods Appraisal Tool (MMAT; Hong et al. 2018) was used to assess the quality of included studies that were classified as ‘quantitative non-randomised’. Narrative synthesis was used to analyse the data.

## Results of McCormack et al (2023)

Seven articles were included in the final synthesis. The studies took place between 2003 and 2020 in the USA (4), Canada (1), Slovenia (1) and Australia (1). There were two prospective cohort studies and five pre-post interventional studies. In total, 267 participants were included ranging in age from nine months to 26 years. The most common medical background was neurological impairment with underlying genetic, cardiac, or metabolic diagnoses. According to the review authors’ quality assessment of included studies, the majority of MMAT criteria were met. However, a number of caveats were also documented regarding target population, outcome measures used, completeness of data and intervention characteristics.

The authors concluded that there was significant heterogeneity within and between the study outcomes. Each of the studies reported on factors related to the use of BD with GI symptoms and growth parameters being the most commonly reported. Nutritional intake, medication use, and progression to oral feed were also discussed.

### Gastrointestinal Symptoms

Two cohort studies found statistically significant improvement in relation to overall GI function, GOR/reflux, nausea, frequency of vomiting, abdominal pain, and diarrhoea, and one of these studies found statistically significant improvement for gas and constipation. One pre-post study found statistically significant improvement in feeding discomfort and prevalence of vomiting. Three other pre-post studies had mixed findings regarding upper GI symptoms, GOR/reflux, constipation, and gagging/retching, and it was unclear whether these findings were statistically significant. One pre-post study found no change in stool consistency and frequency. For further details refer to Table 1.

### Medication use

A cohort study found statistically significant improvement in the use of pro-motility/pro-kinetic medication, but no change in the use of acid-suppressants, laxatives, or anti-diarrhoeal medication. Pre-post studies revealed mixed findings regarding the use of GI related medications, acid-suppressants, and laxatives, but no change in pro-motility/pro-kinetic medication use, as demonstrated in Table 2.

### Growth parameters

While the cohort studies found increases in both the BD and CF groups in terms of fat percentage calculated from triceps skinfold thickness, weight for age/weight, and BMI, these increases were statistically significantly higher in the CF group compared to the BD group. Findings of a pre-post study regarding weight were mixed and one other found no change in BMI. One cohort study found no differences in terms of incidence of malnutrition. While a pre-post study identified a statistically significant increase in height, a cohort study found no change. Additionally, a pre-post study found statistically significant increase in the proportion of patients with triceps skinfold thickness. Table 3 includes further details.

### Transition to BD and transition from BD tube feeding to oral feeding

Three pre-post studies found transition to BD in those who continued tube feeding feasible in the majority of their sample. In these same studies, a small number of patients transitioned from BD to full oral feed. Increased oral intake was found in two participants in a pre-post study and in just over half of the participants of two other pre-post studies.

### Nutritional intake and deficiencies

Four studies investigated the nutrient composition of the feed and three studies (n=97) examined biochemical markers of nutritional deficiencies ad detailed in Table 5.

#### Calorie intake

One cohort and one pre-post study found no difference in calorie intake between BD and CF. One cohort study found that those on BD consumed 40% higher calories per kilogram of body weight than those in the CF group. One pre-post study identified that participants required a 50% greater calorie intake from BD compared to CF to maintain stable BMI.

#### Macronutrients

One cohort study found no significant difference between groups in terms of overall macronutrients. Another identified that participants in the BD group received higher amounts of protein and lower amounts of carbohydrate from BD but found no significant difference in fat intake between the groups. Both cohort studies found higher amounts of fibre in BD.

#### Micronutrients

One pre-post study reported that overall micronutrient composition with BD was superior to CF with the exception of vitamin D, although it is unclear what this means in terms of statistical significance. One cohort study and one pre-post study found insufficient levels of vitamin D in BD.

#### Nutritional deficiencies

One cohort and two pre-post studies, used biochemistry to determine nutritional deficiencies and found no evidence of such in the participants studied.

### Complications/adverse events due to the use of BD

Few participants were found to have experienced any mild adverse events. No cases of severe allergic reaction, life-threatening adverse event, foodborne infection, aspiration pneumonia, or gastrostomy malfunctioning/blocking were identified. Reports showed that a total of three participants discontinued the use of BD as a result of symptom worsening or mild adverse events.

## Commentary

### Critical appraisal of the review by McCormack et al (2023)

Based on the Joanna Briggs Institute (JBI) Critical Appraisal Tool for Systematic Reviews and Evidence Syntheses (Aromataris et al. 2015), the study by McCormack et al (2023) satisfies only five out of the 11 criteria (Table 7). Firstly, the review question was not clearly and explicitly stated and as a result, the inclusion criteria was missing some of the key elements. With a poorly defined inclusion criteria, such as comparator group and age in this case, it is difficult to judge study selection and their combinability. Indeed, the review synthesised studies that were not comparable in design (studies with and without comparator group), which makes interpretation of the findings difficult and limits applicability of the results. Additionally, it was unclear who performed the critical appraisal making it difficult to understand how reliable the findings of the included studies were. Moreover, only one researcher carried out data extraction, which may have led to systematic errors and inappropriate conclusions. Finally, publication bias was not considered, which may lead to overestimation of intervention effectiveness.

Due to the above limitations, the quality issues of included studies (e.g. selection bias, lack of control group, recall bias, use of different measures, variable timescales) and the small sample size studied, the evidence regarding the benefits of BD presented in this review is limited.

The review has demonstrated that, while GI symptoms vary and the included studies covered several different GI-related outcomes, overall, there is some evidence that the use of BD may potentially decrease undesirable GI symptoms. Findings regarding nutritional intake was mixed but there was some consistency in terms of higher fibre intake with BD, which has been found beneficial for bowel function and overall health (Hoisak et al. 2022). Additionally, transitioning to BD was feasible for a high percentage of participants where this was investigated. It is encouraging that findings of this review suggest that the use of BD does not appear to represent any harm as complications were found to be few and mild in nature. However, the vitamin D content of blended foods was found to be inferior to CFs in two of the studies which indicates that additional supplementation of this nutrient may be required. Evidence regarding other outcomes investigated by the review were too inconsistent to yield meaningful conclusions.

Research in this area remains in its infancy and further research is needed to provide quantifiable evidence. Nevertheless, BDA members and nurses increasingly cite anecdotal evidence in support of BD due to its potential to improve well-being, in addition to facilitating family cohesion, a sense of nurturing, and normalisation of feeding through a tube (Durnan, 2018). In 2019 the BDA Policy Statement (2019) was updated to allow members to recommend the practice to patients/families and the updated BDA Toolkit (2021) provides best practice guidance for dietitians and healthcare professionals to ensure effective, equitable and quality nutritional advice and care, and is now actively referenced in the nursing literature (Bremner et al. 2022). Stronger research evidence regarding benefits and complication in addition to robust clinical and social care policies are required to support this tube feeding method.

### Recommendations for future research

While RCTs are the gold standard for identifying intervention effectiveness, this population presents research challenges due to diverse clinical backgrounds. Studies with larger numbers of participants with similar characteristics using a *Core Outcome Set (COS) of measures* (Kirkham 2022) and validated tools would greatly improve the body of research in BD facilitating a more robust synthesis of the evidence. However, no agreed core outcome set exists yet in this area.

Furthermore, based on the findings of McCormack et al (2023) regarding BD’s potential to reduce medication use, future research in this area would be beneficial. Additionally, while the use of multiple different measures has been recommended to monitor nutritional status, it has been debated whether these indicate optimal growth (Oftedal et al. 2025) warranting the need for more investigation. Similarly, consideration must be given to the time period for review and monitoring as time may be a moderating factor in terms of the benefits and complications of BD. Working within the complexity of the underlying medical conditions of this population, changes, either dietary or otherwise, are often better managed with small adjustments in variable factors.

## CPD reflective questions

- Why are families continuing to choose blended diet?
- What conclusions can be drawn from narrative research with differing results?
- Is using Blended Diet a realistic option for all tube feeders?

## Figures and Tables

**Table 1:** Findings regarding gastrointestinal symptoms

Outcomes measured	Hron et al 2019 Cohort study Total n=70 BD n=42 CF n=28	Chandrasekar et al (2022) Cohort study Total n=41 BD n=21 CF n=20	Gallagher et al (2018) Pre-post study Total n=20 Completed n=17^1^	Kernizan et al (2020) Pre-post study Total n=35	Batsis et al (2020) Pre-post study Total n=23	Pentiuk et al (2011) Pre-post study Total n=33
Upper GI symptoms	N/A	N/A	N/A	N/A	Symptoms (n=21) -* (n=20)	N/A
Overall GI function	-*	-*	N/A	N/A	N/A	N/A
GOR/Reflux	-*	-*	N/A	Symptoms (n=33) **-** (n=14) **=** (n=18) **!** (n=1)	N/A	N/A
Nausea	-*	-*	N/A	N/A	N/A	N/A
Vomiting (frequency)	-*	-*	N/A	N/A	N/A	N/A
Vomiting (prevalence)	N/A	N/A	-*	N/A	N/A	N/A
Abdominal pain	-*	-*	N/A	N/A	N/A	N/A
Diarrhoea	-*	-*	N/A	N/A	N/A	N/A
Gas	N/A	-*	N/A	N/A	N/A	N/A
Constipation	N/A	-*	N/A	Symptoms (n=10) - (n=3) **!** (n=1)	!* (n=5)	N/A
Feeding discomfort	N/A	N/A	-* (n=17)	N/A	N/A	N/A
Stool consistency	N/A	N/A	**=**	N/A	N/A	N/A
Stool frequency	N/A	N/A	**=**	N/A	N/A	N/A
Gagging / Retching	N/A	N/A		N/A	N/A	Symptoms (n=33) - (n=17) 76-100% reduction
						- (n=24) ≥50% reduction

**BD**: blended diet; **CF:** commercial formula; -*: statistically significant decrease or statistically significantly difference between the groups in favour of the BD group; **-**: decrease but statistical significance unclear; !*: statistically significant deterioration; **!**: deterioration but statistical significance unclear; **=:** no statistically significant difference between groups or change over time; **N/A**: not applicable as outcome not measured or not reported; ^1^unclear why number of participants reduced; **n**: number of participants (where figures available).

**Table 2:** Findings regarding medication use

Medication type	Chandrasekar et al (2022) Cohort study Total n=41 BD n=21 CF n=20	Gallagher et al (2018) Pre-post study Total n=20 Completed n=17	Kernizan et al (2020) Pre-post study Total n=35	Batsis et al (2020) Pre-post study Total n=23
GI related medication unspecified	N/A	N/A	Medication use (n=33) **-** (n=13) **!** (n=3)	N/A
Acid-suppressant	**=**	-*	N/A	**=**
Pro-motility / Pro-kinetic	-*	**=**	N/A	**=**
Laxatives (including stool softeners)	**=**	! *	**-** (n=3) **!** (n=1)	N/A
Anti-diarrhoeal	**=**	N/A	N/A	N/A

**BD:** blended diet; **CF:** commercial formula; -*: statistically significant decrease/discontinued medication use or statistically significant difference between groups in favour of BD; -: decrease but statistical significance unclear; =: no statistically significant change over time or no statistically significant difference between groups; !*: statistically significant increase or medication uptake; **!**: increase or medication uptake but statistical significance unclear **n:** number of patients (where figures available); **N/A:** not applicable as outcome not measured or not reported.

**Table 3:** Findings regarding growth parameters

Outcomes measured	Orel et al (2017) Cohort study Total n=37 BD n=20 CF n=17	Chandrasekar et al (2022) Cohort study Total n=41 BD n=21 CF n=20	Gallagher et al (2018) Pre-post study Total n=20 Completed n=17	Kernizan et al (2020) Pre-post study Total n=35	Batsis et al (2020) Pre-post study Total n=23	Pentiuk et al (2011) Pre-post study Total n=33
Proportion of patients with Triceps Skinfold Thickness <5^th^ percentile	N/A	N/A	+*	N/A	N/A	N/A
Fat% calculated from Triceps Skinfold Thickness	+* BD & CF Significantly higher increase in CF groups	N/A	N/A	N/A	N/A	N/A
Weight for age / Weight	+* BD & CF Significantly better improvemen t in CF group	N/A Significantly higher weight in CF group	N/A	N/A	=	+* (n=29) **=** (n=4)
Height for age / Height	N/A	**=**	N/A	N/A	+*	N/A
BMI	**+** BD & CF Significantly higher increase in CF group	N/A Significantly higher in CF group	N/A	=	N/A	N/A
Incidence of Malnutrition	N/A	**=**	N/A	N/A	N/A	N/A

**BD:** blended diet; **CF:** commercial formula; +* = statistically significant increase or statistically significant difference between groups; **+:** increase or difference between groups in favour of the BD group, but statistical significance unclear; =: no statistically significant change over time or no statistically significant difference between groups; **n:** number of participants (where figures available); **N/A:** not applicable as outcome not measured or not reported.

**Table 4:** Findings regarding transition to BD and transition from BD tube feeding to oral feeding.

Outcomes measured	Gallagher et al (2018) Pre-post study Total n=20 Completed n=17	Kernizan et al (2020) Pre-post study Total n=35	Batsis et al (2020) Pre-post study Total n=23	Pentiuk et al (2011) Pre-post study Total n=33
Feasibility of transition to BD in those who continued tube feeding	Feasible in 90% (n=17/19)	Feasible in 91% (n=29/32)	Feasible in 86% (n=18/21)	N/A
Transition from BD to oral feeding	5% (n=1)	n=3	9% (n=2/23)	N/A
Increased oral intake	N/A	n=2	53%	57%

**BD**: blended diet; **n**: number of participants (where figures available); **N/A:** not applicable as outcome not measured or not reported.

**Table 5:** Findings regarding nutritional intake and deficiencies.

Outcome measured	Hron et al (2019) Cohort study Total n=70 BD n=42 CF n=28	Chandrasekar et al (2022) Cohort study Total n=41 BD n=21 CF n=20	Gallagher et al (2018) Pre-post study Total n=20 Completed n=17	Kernizan et al (2020) Pre-post study Total n=35	Batsis et al (2020) Pre-post study Total n=23	Pentiuk et al (2011) Pre-post study Total n=33
Calorie intake	**=**	N/A	N/A	**=**	N/A	N/A
Calorie intake per kilogram of body weight	N/A	+*	N/A	N/A	N/A	N/A
Calories required with BD compared to CF to maintain stable BMI	N/A	N/A	50% higher	N/A	N/A	N/A
Macronutrie nts	**=**	N/A	N/A	N/A	N/A	N/A
Protein intake	N/A	**+**	N/A	N/A	N/A	N/A
Carbohydrat e intake	N/A	**-**	N/A	N/A	N/A	N/A
Fat intake	N/A	**=**	N/A	N/A	N/A	N/A
Fibre intake	+*	+*	N/A	N/A	N/A	N/A
Micronutrie nts	N/A	N/A	**+** (except for vitamin D)	N/A	N/A	N/A
Vitamin D	**!**	N/A	**!**	N/A	N/A	N/A
Evidence of deficiencies from blood tests	N/A	None	N/A	N/A	None	None^1^

**BD**: blended diet; **CF:** commercial formula; **BMI**: body mass index; +*: statistically significant increase or statistically significant difference between groups in favour of BD; +: increase or higher in BD group but statistical significance unclear; -*: statistically significant decrease or statistically significantly lower in BD group; -: decrease or lower in BD group but statistical significance unclear; **=:** no statistically significant difference between groups or no statistically significant change over time; **!:** insufficient amount; **n:** number of participants (where figures available); ^**1**^only subset tested; **N/A:** not applicable as outcome not measured or not reported.

**Table 6:** Findings regarding complications/adverse events due to the use of BD.

Outcomes measured	Gallagher et al (2018) Pre-post study Total n=20 Completed n=17	Kernizan et al (2020) Pre-post study Total n=35	Batsis et al (2020) Pre-post study Total n=23	Pentiuk et al (2011) Pre-post study Total n=33
Mild adverse events (rash, abdominal pain, diarrhoea, reflux?)	N/A	n=6/33	N/A	N/A
Adverse events	None	N/A	N/A	None
Severe allergic reactions	N/A	None	N/A	N/A
Life-threatening adverse events	N/A	None	N/A	N/A
Foodborne infections / Hospitalisation due to foodborne infection	N/A	None	None	N/A

**N/A:** not applicable as outcome not measured or not reported; **n**: number of participants (where figures available).

**Table 7:** Critical appraisal using the JBI critical appraisal checklist for systematic reviews and research syntheses (Aromataris et al, 2015).

JBI critical appraisal checklist items	Responses
1. Is the review question clearly and explicitly stated?	**No**
2. Were the inclusion criteria appropriate for the review question?	**No** (studies without a comparator group were also included and criteria for age was unclear)
3. Was the search strategy appropriate?	**Yes**
4. Were the sources and resources used to search for studies adequate?	**Yes**
5. Were the criteria for appraising studies appropriate?	**Yes**
6. Was critical appraisal conducted by two or more reviewers independently?	**No** (unclear who performed the critical appraisal)
7. Were there methods to minimize errors in data extraction?	**No** (only one researcher carried out data extraction)
8. Were the methods used to combine studies appropriate?	**Yes**
9. Was the likelihood of publication bias assessed?	**No**
10. Were recommendations for policy and/or practice supported by the reported data?	**No** (recommendations were not made)
11. Were the specific directives for new research appropriate?	**Yes**

## Key Points

- Research on blended diet for tube-fed children has been identified as a top priority for clinicians and families due to its growing popularity.
- Emerging evidence suggests GI improvements may be seen.
- Few participants suffered any adverse events following introduction of blended diet and in those who did only minor issues were encountered.
- Further research is needed to identify the effects of blended diet.
